# A Retrospective Cohort Study of Distal Biceps Brachii Tendon Rupture and Subsequent Repair Methods: A Comparison of Two Combined Treatment Pathways

**DOI:** 10.7759/cureus.116557

**Published:** 2026-09-19

**Authors:** Niall E Hill, Peadar A Mac Suibhne, Ryan J Browne, Carla J Foxworthy, Adam Reaney, Frank S Davis, Awen Iorwerth, Dai Morgan

**Affiliations:** 1 Orthopedics and Trauma, University Hospital Wishaw, Lanarkshire, GBR; 2 Orthopedics and Trauma, Bon Secours Hospital Tralee, Tralee, IRL; 3 Surgery, Great Western Hospital, Swindon, GBR; 4 General Surgery, University Hospital Hairmyres, Lanarkshire, GBR; 5 Orthopedics and Trauma, Cardiff University, Cardiff, GBR; 6 Orthopedics and Trauma, South West Orthopaedics and University of Exeter, Exeter, GBR; 7 Orthopedics and Trauma, Cwm Taf Morgannwg University Health Board, Cardiff, GBR

**Keywords:** biceps tendon repair, complication rates, distal biceps tendon, retrospective cohort study, surgical technique, tendon rupture, upper limb surgery

## Abstract

Background

Distal biceps brachii tendon rupture is an uncommon injury that is predominantly caused by excessive eccentric force to a flexed elbow. Operative repair is generally preferred to restore function, with a variety of surgical fixation techniques and postoperative rehabilitation protocols currently employed but with no clear consensus regarding the optimal approach. Comparative evidence evaluating surgical techniques remains limited.

Objective

The objective of this study is to compare two combined treatment pathways for distal biceps tendon repair using postoperative complications such as rerupture rates to compare postoperative outcomes to complication profiles.

Methods

All patients undergoing distal biceps repair in a single center in Wales, United Kingdom, between 2018 and 2022 were included. Patients were operated on by one of two surgeons. Information was gathered from electronic patient records and radiographic images. Demographics including age, gender, hand dominance, occupation, the mechanism of injury, and surgical intervention were recorded. Clinic letters were reviewed to determine the incidence of postoperative function and complications.

Results

Forty-four men were included with a median age of 45 years. All patients reported eccentric contraction as their mechanism of injury. The injury affected the dominant upper limb in all 44 patients (100%). The median time to surgery after imaging was 24 days. In the Surgeon 1 cohort, lateral cutaneous nerve (LCN) numbness was documented in 15 patients (50.0%) and elbow stiffness in six patients (20.0%). In the Surgeon 2 cohort, LCN paresthesia occurred in four patients (28.6%), stiffness in three patients (21.4%), and superficial radial nerve (SRN) paresthesia in two patients (14.3%). One patient required a revision of their biceps tendon repair (2%). All but one patient in each cohort achieved complete functional resolution by three months postoperatively.

Conclusions

The operative management of distal biceps rupture in this unit demonstrates similar postoperative outcomes from both treatment pathways.

## Introduction

Distal biceps brachii ruptures are an uncommon injury of the upper limb, typically resulting from trauma [1]. The incidence of this pathology is approximately 1.2-2.55 per 100,000 [2-4]. Active men in the fourth to sixth decades of life are more likely to be affected by this condition, with the dominant arm particularly susceptible to injury [5-7]. The most common mechanism for this condition is when excessive eccentric force is applied to an elbow that is flexed, leading to pain and significant loss in supination power and elbow flexion [1,2,8]. Occupation is an important demographic factor in distal biceps rupture, as physically demanding jobs may predispose individuals to injury, while the associated loss of strength and function can have a substantial impact on manual labor occupations [1]. Activities such as weightlifting and contact sports have also been associated with this injury [2]. The cosmetic appearance of this injury is unacceptable to many patients, with the classic "Popeye" deformity seen in many cases of distal biceps tendon rupture where the excessive retraction of the muscle belly occurs, presenting as a bulbous mass [1].

Distal biceps tendon rupture may be managed operatively or nonoperatively. The latter typically results in a persistent reduction of flexion and supination strength, leading to significantly reduced endurance [8,9]. A biomechanical study found the mean loss in supination to be 40% and flexion 30% with nonoperative management; hence, operative management is very often desired in patients for whom surgical intervention is possible [8,10].

Operative treatment, however, is not devoid of risk. There are many associated risks to both biceps tendon repair and reconstruction including, but not limited to, cutaneous nerve neuropraxias, heterotopic ossification, tendon rerupture, and stiffness [6,11].

This study aimed to compare two distinct treatment pathways for distal biceps tendon repair, incorporating different surgical and postoperative management approaches performed by two consultant orthopedic surgeons at a single center, and to assess the postoperative outcomes and complication profiles associated with each pathway.

## Materials and methods

This was a retrospective cohort study of adult patients (>18 years of age) diagnosed with a distal biceps tendon rupture and managed at a district general hospital in the Cwm Taf Morgannwg University Health Board, Wales, United Kingdom, between April 2018 and December 2022. The diagnosis was established clinically, with or without secondary radiological confirmation via ultrasound or magnetic resonance imaging. Patients were excluded if clinical records, operative reports, or follow-up data were insufficient to permit a reliable retrospective evaluation. Institutional review board/ethics committee approval was not required for this retrospective cohort study involving anonymized patient data, as determined in accordance with institutional policies.

Preoperatively, demographic and clinical variables were extracted from electronic health records. Recorded parameters included patient age, sex, injured laterality, hand dominance, mechanism of injury, date of injury, history of steroid use (past or current anabolic-androgenic steroid or corticosteroid exposure), date of diagnostic ultrasound scan, and corresponding radiological findings. Intraoperative metrics recorded for all cases included the date of surgery, primary operating surgeon, surgical approach, and total tourniquet time in minutes.

All surgical procedures were performed by one of two consultant orthopedic surgeons utilizing two different operative, postoperative, and rehabilitation techniques as outlined in Table 1. Surgeon 1 operated on 30 patients (n = 30) using a single "Lazy-S" anterior incision. Tendon fixation was achieved using Orthocord® suture (DePuy Synthes/Johnson & Johnson, New Brunswick, NJ) tied twice onto the dorsal radius without hardware augmentation. Postoperative immobilization for this cohort consisted of an above-elbow plaster of Paris cast maintained for six weeks. Surgeon 2 operated on 14 patients (n = 14) using a single "Lazy-S" anterior incision. Tendon fixation was completed using FiberLoop® suture (Arthrex, Inc., Naples, FL) and a SutureButton® (Arthrex, Inc.) cortical construct, augmented with an interference tenodesis screw. Postoperative immobilization for this group utilized a hinged range-of-motion elbow brace for six weeks.

**Table 1:** Comparison of surgical techniques and postoperative protocols by surgeon. All registered trademarks belong to their respective owners. n: number of patients

Operating Surgeon	Method of Fixation	Suture Button	Augment	Incision	Postoperative Immobilization Method	Rehabilitation Protocol
Surgeon 1 (n = 30)	Orthocord® ×2 tied into the dorsal radius	None	None	Single "Lazy-S"	Plaster of Paris for six weeks	Fully immobilized for six weeks
Surgeon 2 (n = 14)	FiberLoop® and SutureButton®	Yes	Tenodesis screw	Single "Lazy-S"	Range-of-movement brace for six weeks	Early range of movement from day 1 postoperatively with gradual increase in range

Postoperative variables collected during routine follow-up included the method and duration of immobilization, issues or compliance related to immobilization, rehabilitation protocol, duration to return to work, and the date of final clinic discharge. Early complications (occurring within six weeks postoperatively) and long-term complications, such as cutaneous nerve neuropraxias, stiffness, and repair failure or rerupture, were documented. All patient data were anonymized and stored on an encrypted drive on National Health Service-approved devices in compliance with information governance regulations.

## Results

Forty-four patients who underwent operative treatment for a distal biceps brachii rupture were included in this study. The median age of the cohort was 45 years, with a modal peak in the fifth decade of life as depicted in Figure 1. All included patients were men (100%). One patient was excluded due to inaccessible digital health records. The injury affected the dominant upper limb in all 44 patients (100%). All surgical procedures were performed by one of two consultant orthopedic surgeons within the unit, designated as Surgeon 1 (n = 30) and Surgeon 2 (n = 14). Only one patient required revision surgery. Anabolic steroid misuse was documented in nine patients (20.5%) who reported current or past use. Percentage differences should be interpreted as descriptive only.

**Figure 1:**
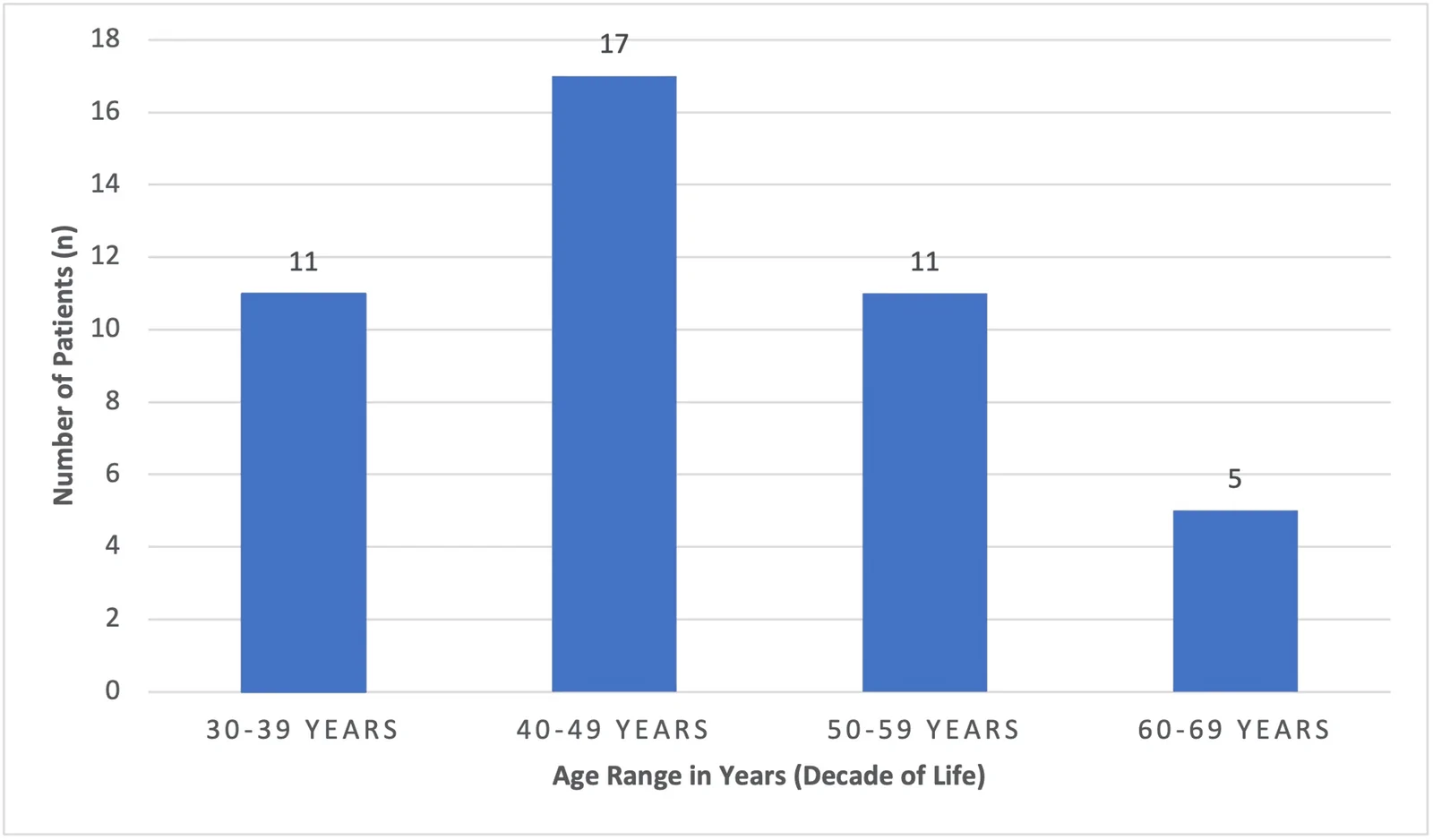
Age distribution of distal biceps brachii tendon ruptures in the study cohort. Distribution of distal biceps brachii tendon ruptures by age decade across the patient cohort (N = 44). Peak incidence occurred in the fifth decade of life (40-49 years of age).

All patients reported an eccentric contraction mechanism of injury. Sports participation was the index event in 17 patients (38.6%), with the remaining 27 patients (61.4%) sustaining the injury during heavy lifting in a non-sporting or occupational context. The median time from ultrasound evaluation to surgical intervention was 24 days. Seventy-three percent of the cohort were employed in manual labor occupations. The baseline demographic and clinical characteristics of the overall cohort and surgeon subgroups are summarized in Table 2.

**Table 2:** Demographic and baseline clinical characteristics of the patient cohort (N = 44). Data are presented as mean ± standard deviation (SD) for continuous variables and as frequency (n) with percentage (%) for categorical variables. All patients in the study were men (100%) and presented with acute distal biceps tendon rupture resulting from an eccentric load mechanism. Subgroup comparisons are provided for procedures performed by Surgeon 1 (n = 30) and Surgeon 2 (n = 14). N, total cohort size; n, subgroup sample size

Parameter	Total Cohort (N = 44)	Surgeon 1 Cohort (n = 30)	Surgeon 2 Cohort (n = 14)
Age (years), mean ± SD	49.2 ± 9.2	49.8 ± 9.0	48.0 ± 9.8
Age range (years)	34-68	34-68	36-66
Sex (male/female), n (%)	44/0 (100%)	30/0 (100%)	14/0 (100%)
Primary injury biomechanics, n (%)			
Acute eccentric contraction	44 (100%)	30 (100%)	14 (100%)
Injury setting/context, n (%)			
Sports-related activity	17 (38.6%)	12 (40.0%)	5 (35.7%)
Non-sports/daily activities	27 (61.4%)	18 (60.0%)	9 (64.3%)
Anabolic steroid use (past/current), n (%)	9 (20.5%)	5 (16.7%)	4 (28.6%)

Postoperative complications varied between the surgical cohorts, as seen in Figure 2. In the Surgeon 1 cohort, lateral cutaneous nerve (LCN) numbness was documented in 15 patients (50.0%) and elbow stiffness in six patients (20.0%). In the Surgeon 2 cohort, LCN paresthesia occurred in four patients (28.6%), stiffness in three patients (21.4%), and superficial radial nerve (SRN) paresthesia in two patients (14.3%). All but one patient in each cohort achieved complete functional resolution by three months postoperatively, which was defined as the subjective absence of pain, stiffness, and significant functional deficit.

**Figure 2:**
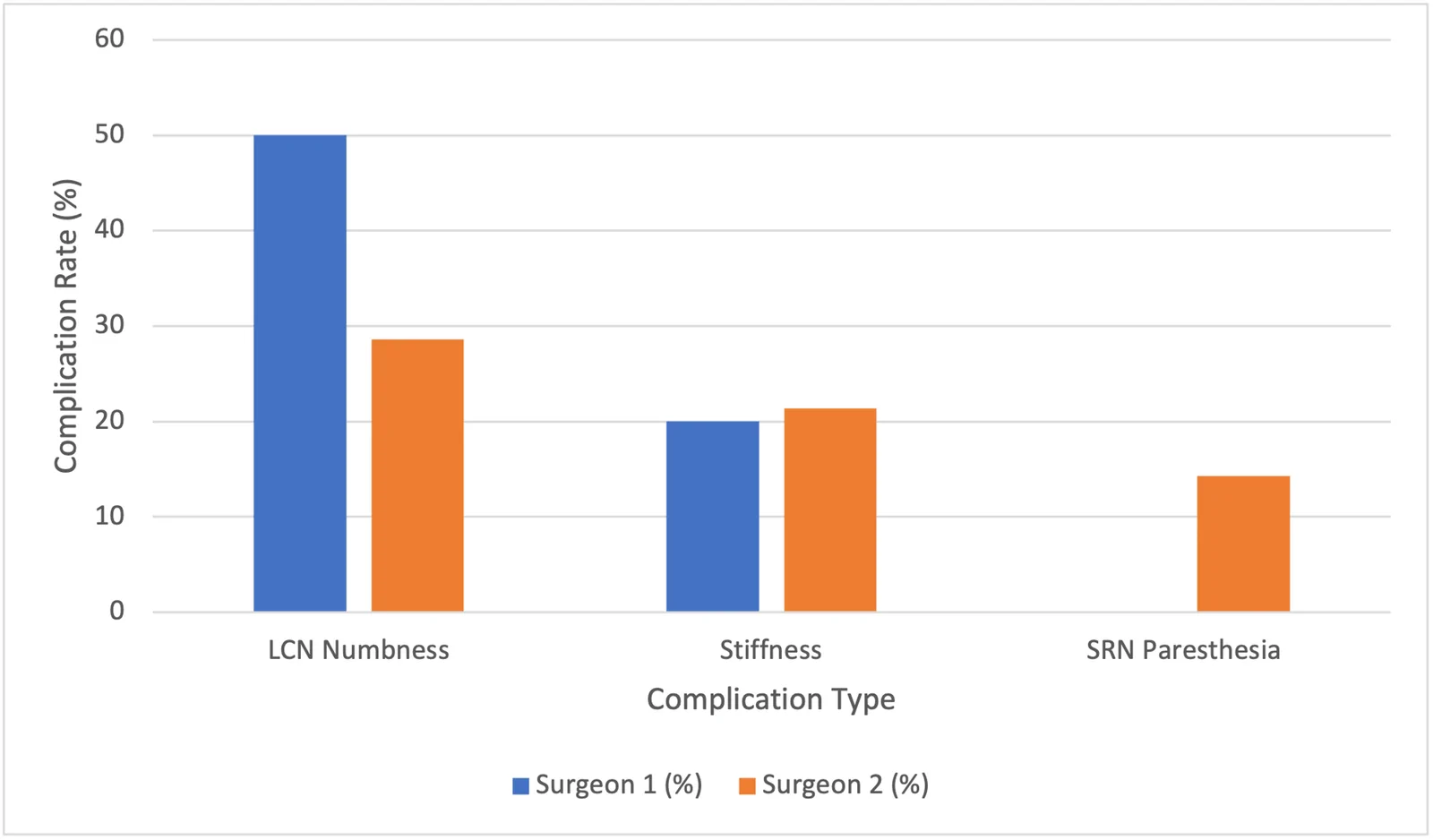
Postoperative complication rates between surgical cohorts. Comparison of early and late postoperative complications in patients operated on by Surgeon 1 (n = 30) utilizing an Orthocord® suture repair and Surgeon 2 (n = 14) utilizing a SutureButton® with tenodesis screw augmentation. LCN, lateral cutaneous nerve; SRN, superficial radial nerve

## Discussion

Overview and key findings

In this retrospective cohort study comparing two distal biceps tendon repair and postoperative protocols, both treatment pathways demonstrated excellent structural integrity with low revision rates and high rates of complete neurological recovery. However, key differences emerged regarding specific nerve complications and patient demographics.

Rerupture rates and repair stability

This study identified one patient who required a revision of their biceps tendon repair (2%). This figure is less than other single-incision biceps repair studies in the literature, which found the rerupture rate to lie between 4% and 6% [12,13]. It is similar to the rerupture rate of 1.4% found in a large systematic review that assessed complications of distal biceps tendon repair [11]. It has often been found that reruptures may not be solely attributable to fixation technique, with falls and noncompliance as examples of other potential causes [13]. It has been hypothesized that age and biceps rupture are dependently associated due to an inverse relationship that exists between age and the tensile strength of tendons [5,14]. The most common decade for biceps brachii rupture in this cohort was in the fifth decade of life. There are numerous other factors that determine susceptibility to tendon ruptures, including genetic, structural, overuse, and lifestyle factors [5,15].

Nerve complications and bracing mechanisms

The most notable difference in postoperative complications between cohorts was the absence of superficial radial nerve (SRN) involvement in the Surgeon 1 cohort. The postoperative immobilization brace may offer an explanation as to why SRN paresthesia was observed exclusively in patients operated on by Surgeon 2. Transient SRN compression is a recognized complication associated with functional bracing [7,11].

Neurapraxias are a recognized complication of distal biceps tendon repair [12,16]. Although rare, the compression of other forearm nerves, such as the median nerve, has also been documented [17]. These complications occur more frequently when distal biceps tendon ruptures are repaired using a single-incision approach, which was utilized by both surgeons in the present study [7,13]. The rate of short-term neurapraxia in the Surgeon 1 cohort (50.0%) was slightly higher than rates reported in comparable single-incision studies, such as one evaluation of distal biceps repairs that reported a complication rate of 40% [13]. However, all but one patient in each cohort experienced a complete resolution of neurapraxia by three months postoperatively.

Demographic factors and occupational demand

Seventy-three percent of the cohort were employed in manual labor occupations, substantially higher than the 29% reported in similar cohorts [3,14,18]. This heavy occupational demand may account for increased baseline strain on the repair and heightened vulnerability to short-term postoperative stiffness or neurapraxia, despite the low overall rerupture rate observed.

Study limitations

There are several limitations to this study. First, as a retrospective cohort study lacking a control arm, it provides level IV evidence. Second, there was an absence of validated patient-reported outcome measures (PROMs), such as the EuroQol 5-Dimension (EQ-5D) scale, which could have provided greater insight into patients' functional outcomes, health-related quality of life, and overall satisfaction following distal biceps tendon repair. Third, the cohort exhibited a complete male propensity (100% male). While distal biceps brachii ruptures occur far less frequently in women, these results may not be generalizable to female patients. Furthermore, because data were collected from a single orthopedic unit with a modest sample size (N = 44), the findings may not fully reflect the broader patient population. Additionally, reliance on follow-up clinical documentation to identify complications introduces potential reporting bias, as minor or transient complications may not have been explicitly recorded by the treating clinician. Strength testing postoperatively was not standardized nor range of motion measurements, which limits the conclusions that may be drawn between both cohorts. The unequal distribution between cohorts (Surgeon 1: n = 30 versus Surgeon 2: n = 14) limits the statistical power of direct subgroup comparisons, meaning percentage differences in this study should be interpreted with caution. As the surgeon, fixation method, and postoperative rehabilitation protocol differed between the two cohorts, the study evaluated the outcomes associated with each overall treatment pathway rather than the independent effect of any individual component.

Finally, the reported rate of anabolic steroid misuse may underestimate true prevalence due to underreporting. The nondisclosure of anabolic androgenic steroid use is well-documented in orthopedic literature [19,20]. Given that steroid misuse is a major risk factor for tendon rupture, patient reluctance to disclose use may have introduced unmeasured confounding within the nonuser subgroup. While objective screening such as urine testing could identify active users, it lacks the sensitivity to detect past use occurring months or years prior to injury [20].

## Conclusions

Although limited by a modest sample size from a single orthopedic unit, our findings demonstrate comparable overall success and favorable postoperative outcomes between both cohorts. Both operative methods achieved the successful resolution of distal biceps brachii tendon ruptures, with only one patient (2.3%) requiring revision surgery. However, notable differences in neurological complication profiles were observed: lateral cutaneous nerve paresthesia was more frequent in the Surgeon 1 cohort, whereas superficial radial nerve compression occurred exclusively in the Surgeon 2 cohort, which may possibly be attributable to postoperative functional bracing. Larger, multicenter studies are warranted to further compare specific neurapraxia incidence and rerupture rates across diverse surgical techniques and rehabilitation protocols.
